# Is a Two-Year Growth Response to Growth Hormone Treatment a Better Predictor of Poor Adult Height Outcome Than a First-Year Growth Response in Prepubertal Children With Growth Hormone Deficiency?

**DOI:** 10.3389/fendo.2021.678094

**Published:** 2021-06-01

**Authors:** Saartje Straetemans, Raoul Rooman, Jean De Schepper

**Affiliations:** ^1^ Department of Pediatric Endocrinology, Maastricht University Medical Center, Maastricht, Netherlands; ^2^ NUTRIM School of Nutrition and Translational Research in Metabolism, Maastricht University, Putte, Netherlands; ^3^ The BElgian Society for PEdiatric Endocrinology and Diabetology (BESPEED), Brussels, Belgium; ^4^ PendoCon bv, Putte, Belgium; ^5^ Department of Pediatric Endocrinology, University Hospital Brussels, Brussels, Belgium; ^6^ Department of Pediatric Endocrinology, University Hospital Ghent, Ghent, Belgium

**Keywords:** growth hormone treatment, growth hormone deficiency, children, growth response, poor adult height outcome

## Abstract

**Objective:**

The first year response to growth hormone (GH) treatment is related to the total height gain in GH treated children, but an individual poor first year response is a weak predictor of a poor total GH effect in GH deficient (GHD) children. We investigated whether an underwhelming growth response after 2 years might be a better predictor of poor adult height (AH) outcome after GH treatment in GHD children.

**Design and methods:**

Height data of GHD children treated with GH for at least 4 consecutive years of which at least two prepubertal and who attained (near) (n)AH were retrieved from the Belgian Register for GH treated children (n = 110, 63% boys). In ROC analyses, the change in height (ΔHt) SDS after the first and second GH treatment years were tested as predictors of poor AH outcome defined as: (1) nAH SDS <−2.0, or (2) nAH SDS minus mid-parental height SDS <−1.3, or (3) total ΔHt SDS <1.0. The cut-offs for ΔHt SDS and its sensitivity at a 95% specificity level to detect poor AH outcome were determined.

**Results:**

Eleven percent of the cohort had a total ΔHt SDS <1.0. ROC curve testing of first and second years ΔHt SDS as a predictor for total ΔHt SDS <1.0 had an AUC >70%. First-year ΔHt SDS <0.41 correctly identified 42% of the patients with poor AH outcome at a 95% specificity level, resulting in respectively 5/12 (4.6%) correctly identified poor final responders and 5/98 (4.5%) misclassified good final responders (ratio 1.0). ΔHt SDS after 2 prepubertal years had a cut-off level of 0.65 and a sensitivity of 50% at a 95% specificity level, resulting in respectively 6/12 (5.5%) correctly identified poor final responders and 5/98 (4.5%) misclassified good final responders (ratio 1.2).

**Conclusion:**

In GHD children the growth response after 2 prepubertal years of GH treatment did not meaningfully improve the prediction of poor AH outcome after GH treatment compared to first-year growth response parameters. Therefore, the decision to re-evaluate the diagnosis or adapt the GH dose in case of poor response after 1 year should not be postponed for another year.

## Introduction

The goal of growth hormone (GH) treatment in a GH deficient (GHD) child is to attain a true catch-up growth, resulting in an adult height (AH) close to target height (1). The pattern of GH induced growth consists of a first phase of accelerated growth, which allows the child to approach its target height in a number of years and is followed by a phase of maintenance growth where height velocity (HV) is normal. Several studies have evidenced that this GH induced growth acceleration diminishes rapidly, which is called the waning effect (2, 3). This waning has been explained by a GH receptor desensitization, but its determinants have been poorly studied in children with GHD.

In clinical practice, the first-year growth response is most often used to evaluate the individual response to GH treatment (4, 5), allowing the early identification of GHD patients who may not respond to a physiological GH replacement and/or are not GHD. However, we recently showed that the currently used first-year growth response and responsiveness parameters have a low sensitivity and/or specificity to predict a suboptimal adult height outcome after long-term GH treatment in prepubertal GHD children (6).

Many issues may negatively influence the first year response to GH treatment, including GH injection problems, an inappropriate GH starting dose, a hidden growth limiting disease, or additional hormonal deficiencies appearing during GH therapy (*e.g.* central hypothyroidism) (7). Correction of these conditions in the second year may result in an improved linear growth during the second year. In addition, a less pronounced waning effect in the second year of GH treatment might also explain why some children with an inadequate first-year growth response do have an adequate AH outcome.

We therefore investigated in prepubertal children with a non-organic GHD: 1) the contribution of the first 2 years of GH therapy to the total height increase, 2) the magnitude and determinants of the waning of the growth response during the second year, and 3) the eventual improvement of poor adult height prediction after two years of GH therapy in comparison with the prediction after one year of GH treatment. We hypothesized that the growth response after 2 years of GH treatment may be a better predictor of poor adult height outcome than the first year response, as in some patients a less pronounced waning in the second year might compensate a failing first year growth response to GH therapy.

## Materials and Methods

### Materials

The auxological data and GH treatment characteristics of children diagnosed with GHD, collected by the members of the BElgian Society for PEdiatric Endocrinology and Diabetology (BESPEED) in a national database, called Belgrow, since 1986 were retrieved. This study was approved by the ethical committee of Brussels Free University and the University Hospital Brussels in Belgium. All subjects gave written informed consent in accordance with the Declaration of Helsinki. In the registry, all data are pseudonymized to comply with rigorous privacy guidelines. Only patients who had been treated exclusively with daily recombinant human GH for at least 4 consecutive years of which at least two were prepubertal and who had attained (near) AH (nAH) were included. The patients were mostly treated in a time period when the dose for GHD in Belgium was fixed to 25 mcg/kg * day. GHD patients with central malformations (*e.g.* anomalies of the pituitary and/or stalk) and patients with idiopathic GHD as well as patients with congenital GHD related to genetic alterations (*e.g.* GH gene mutations) were included, but those with acquired GHD of known cause (related to *e.g.* a brain tumor, brain irradiation, brain trauma) were excluded. Other exclusion criteria were any medication or known medical condition other than GHD that could affect growth, interruption of GH treatment for more than 6 months, and a birth weight and/or birth length below −2 SD. Girls aged ≥12 years and boys aged ≥13 years at the end of the second GH treatment year were excluded. In total, 110 patients (69 males and 41 females) with non-acquired GHD (66 with isolated GHD and 44 with multiple pituitary hormone deficiency) met the inclusion and exclusion criteria.

### Methods

The diagnosis of GHD was made by the treating physician and peer-reviewed by BESPEED members (8). All patients had a peak GH concentration of <10 µg/L after both glucagon and insulin stimulation. Pubertal onset was defined as testicular volumes ≥4ml for boys and Tanner breast stage ≥2 in girls.

Birth weight for gestational age was transformed into SDS, based on the standards of Niklasson et al. (9). The MPH was calculated according to Tanner et al. as follows: (father’s height + mother’s height + 13 for boys/−13 for girls)/2 (10). Height, weight, BMI, and MPH were converted to SDS using Flemish reference data (11).

nAH was defined as the height attained when HV was less than 2 cm/year, calculated over a period of minimum 9 months, and chronological age >17 years in boys and >15 years in girls. nAH SDS was calculated in two different ways, using the Flemish reference data: (1) for the chronological age (CA), (2) for an age of 21 years (A21).

The change in height (ΔHt) SDS was calculated after the first and second prepubertal year of GH therapy, provided that the height data were available within a 9–15 month interval for that year and scaled to respectively 12 and 24 months.

The final outcome of the GH treatment was evaluated by three different methods: (1) nAH, expressed as a height SDS; (2) total ΔHt SDS, calculated as the nAH SDS minus height SDS at start of GH treatment; (3) nAH SDS minus midparental height (MPH) SDS, an index of achieving the genetic height potential. A poor final treatment outcome was defined as total ΔHt SDS <1.0, nAH SDS − MPH SDS <−1.3, and nAH SDS <−2.0

Receiver operating characteristic (ROC) curve analyses were performed for ΔHt SDS after the first and second prepubertal years as a predictor for the defined poor adult height outcome parameters. We have previously published the results of ROC analyses of the first year only, in a cohort overlapping the cohort of this study (6).

### Statistical Analysis

The variables are reported as the median (25–75th percentiles) and mean (± SD). A Shapiro–Wilk test was used to test for the normal distribution. ROC curve analyses were performed to examine the relationship between sensitivity and specificity for the different test parameters and the different outcome parameters. Only pairs with an area under the ROC-curve (AUC) ≥70% were further analyzed. In order to misdiagnose only 5% of good responders, a specificity level of 95% was chosen to calculate the corresponding cut-off values for ΔHt SDS. Linear regression analyses were performed to study the relationship between the growth responses and possible explanatory variables. Significance was considered at the 5% level (p < 0.05). MedCalc^®^ and IBM SPSS Statistics 25^®^ software was used for all statistical analyses.

## Results

### Clinical Characteristics

The background and auxological characteristics of 110 included GHD children (69 males, 41 females) are listed in **Table 1**. GH therapy was initiated at a mean age of 6.2 years and at a median height SDS of −3.47, which was 2.47 SDS below the MPH SDS. The mean GH dose at start was 28 µg/kg.day. The mean duration of GH therapy was 10.2 years, with a mean duration before pubertal onset of 6.2 years. Girls entered puberty spontaneously at a mean age of 11.3 years (n = 35), boys at a mean age of 12.5 years (n = 45). Puberty was hormonally induced at a mean age of 12.9 years in girls (n = 5) and 13.9 years in boys (n = 20). nAH was attained at a mean age of 16.7 years in girls and 18.7 years in boys.

**Table 1:** Characteristics: background, at GH start, after 1^st^ year, after 2^nd^ year, at pubertal onset, at nAH.

	n	median	p25	p75	mean	SD
**Background**						
gestational age, weeks	104	40.0	38.0	40.0	38.5	2.9
birth weight, SDS	103	−0.27	−0.77	0.25	−0.18	0.89
birth length, SDS	93	−0.27	−0.77	0.25	−0.24	0.95
father height, SDS	105	−1.20	−1.80	−0.15	−1.03	1.17
mother height, SDS	105	−0.78	−1.62	−0.27	−0.91	1.16
MPH, SDS	105	1.05	−1.71	−0.45	−0.99	0.95
maximum GH peak, µg/L	110	3.9	2.1	6.7	4.3	2.7
**at start GH treatment**						
age, years	110	6.1	4.6	8.2	6.2	2.3
height, SDS	110	−3.44	−3.99	−2.80	−3.47	0.86
height minus MPH, SDS	105	−2.44	−3.10	−1.75	−2.47	1.11
BMI, SDS	110	−0.42	−1.20	0.41	−0.33	1.11
GH dose, µg/kg.day	110	27.0	24.5	31.1	28.0	5.5
**after first year GH treatment**						
height, SDS	110	−2.36	−2.91	−1.89	−2.42	0.83
Δ height, SDS^a^	110	1.03	0.65	1.40	1.05	0.50
Δ height velocity, cm/year	95	4.8	3.1	7.2	5.2	3.2
height minus MPH, SDS	105	−1.34	−2.03	−0.73	−1.41	0.98
**after second year GH treatment**						
height, SDS	110	−1.92	−2.52	−1.47	−1.95	0.88
Δ height, SDS^b^	110	1.44	0.95	2.01	1.52	0.72
Δ height velocity, cm/year	110	−2.5	−3.6	−1.3	−2.5	1.9
height minus MPH, SDS	105	-0.94	-1.62	-0.28	-0.94	0.97
**at puberty onset**						
age onset spontaneous puberty (females), years	35	11.4	10.6	12.1	11.3	1.0
age puberty induction (females), years	5	13.0	11.8	13.9	12.9	1.0
age onset spontaneous puberty (males), years	45	12.7	12.0	13.1	12.5	1.0
age puberty induction (males), years	20	14.0	13.3	14.2	13.9	1.2
duration GH therapy before puberty, years	105	6.3	4.4	8.2	6.2	2.4
height, SDS	104	-1.52	-2.29	-0.97	-1.52	1.09
Δ height, SDS^c^	104	1.80	1.14	2.65	1.94	1.03
height minus MPH, SDS	99	-0.49	-1.27	0.05	-0.52	1.11
**at nAH**						
age, years	110	17.9	16.9	18.9	18.0	2.2
age, years (females)	41	16.5	15.2	17.8	16.7	1.8
age, years (males)	69	18.3	17.1	19.2	18.7	2.2
age stop GH treatment, years	110	16.5	15.4	17.4	16.4	1.5
age stop GH treatment, years (females)	41	15.2	14.5	16.5	15.5	1.4
age stop GH treatment, years (males)	69	16.8	16.1	17.6	16.9	1.3
growth since stop GH treatment, cm	106	0.5	0.0	1.2	1.3	2.5
duration GH therapy, years	110	10.2	8.2	12.0	10.2	2.4
nAH CA, SDS	110	-1.21	-1.97	-0.39	-1.21	1.12
nAH A21, SDS	110	-1.53	-2.22	-0.67	-1.44	1.14
nAH CA minus MPH, SDS	105	-0.20	-0.72	0.46	-0.19	0.99
nAH A21 minus MPH, SDS	105	-0.42	-0.99	0.22	-0.43	0.98
Δ height (onset puberty until nAH CA), SDS	104	0.26	-0.18	0.81	0.28	0.26
Δ height (onset puberty until nAH A21), SDS	104	0.02	-0.55	0.71	0.05	0.84
total Δ height CA, SDS^d^	110	2.09	1.56	3.00	2.27	1.11
total Δ height A21, SDS^d^	110	1.86	1.18	2.74	2.03	1.16
BMI CA, SDS	95	-0.11	-1.12	0.65	-0.16	1.33
BMI A21, SDS	95	-0.50	-1.53	0.33	-0.46	1.42

GH, growth hormone; MPH, midparental height; BMI, body mass index; nAH, near adult height; SDS, standard deviation score; cm, centimeter; A21, SDS calculated at age 21 years; CA, SDS calculated at chronological age; ^a^gain in height SDS from start until after first-year GH treatment; ^b^gain in height SDS from start until after second year of GH treatment; ^c^gain in height SDS from start of GH treatment until onset puberty; ^d^gain in height SDS from start of GH treatment until nAH.

### Response to GH Treatment During the First Two Years of Treatment

**Figure 1:**
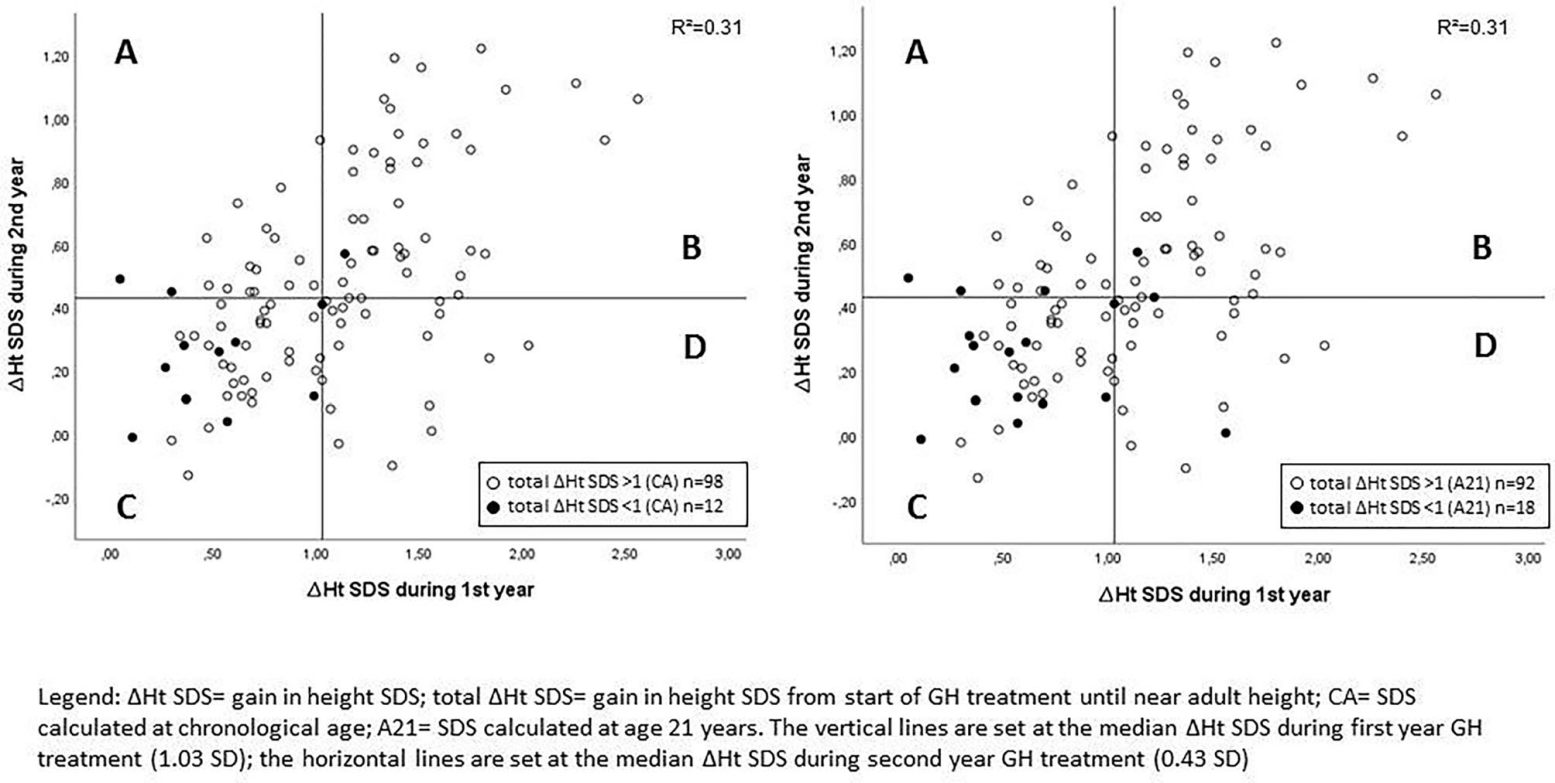
Correlation between Δ height SDS during the first and second GH treatment years with indication of poor final responders. A: lower than median ΔHt SDS during 1st year and higher than median ΔHt SDS during 2nd year; B: higher than median ΔHt SDS during 1st year and higher than median ΔHt SDS during 2nd year; C: lower than median ΔHt SDS during 1st year and lower than median ΔHt SDS during 2nd year; D: higher than median ΔHt SDS during 1st year and lower than median ΔHt SDS during 2nd year.

The median ΔHt SDS after the first treatment year was 1.03, while the median ΔHt SDS during the second year was 0.43 (**Table 1**). **Figure 1** shows the individual data and the correlation between Δ height SDS during the first and second GH treatment years. The ΔHt SDS during the second year correlated moderately (r = 0.553; p < 0.001) with the first year height increase. Patients with a lower than median ΔHt SDS (<1.03 SD) during the first treatment year had a median second year ΔHt SDS of 0.29 SD, which was 0.33 SD lower than the first year; their median ΔHt SDS after 2 years was 0.95. In contrast, patients with a higher than median first-year ΔHt SDS (>1.03) had a median second year ΔHt SDS of 0.57 SD, which was 0.77 SD lower than the first year; their median ΔHt SDS after 2 years was 2.01. Of the 55 patients with a higher than median first-year ΔHt SDS, 19 had a lower than median second-year response (shown in quadrant D in **Figure 1**), while 17/55 patients with a lower than median first-year ΔHt SDS had a higher than median second year increase (shown in quadrant A). Only 4/110 patients had a second year ΔHt SDS that was higher than the first year ΔHt SDS.

### Determinants of the Waning Effect During the Second Year

The first year ΔHt SDS correlated negatively with maximum GH peak in the GH stimulation tests, age at start, height minus MPH SDS at start, height SDS at start, and correlated positively with BMI SDS at start, mid parental height SDS and GH dose at start (**Table 2**). Whereas the height SDS increase in the second year correlated positively with first-year ΔHt SDS and negatively with maximum GH peak, height minus MPH SDS at start, height SDS at start, and age at start. The waning effect, calculated by the difference between ΔHt SDS in the second year and ΔHt SDS in the first year, was positively correlated with first-year ΔHt SDS, height SDS after the first and second years, BMI SDS at start, and correlated negatively with age at start, height minus MPH at start, maximum GH peak, and height SDS at start.

**Table 2:** Linear regression analyses for the prediction of first and second year ΔHt SDS and the waning effect during the second GH treatment year in prepubertal GHD patients.

predictors	R²	p-value	correlation pos/neg
***first-year ΔHt SDS^a^***			
maximum GH peak	0.302	<0.001	−
age at start	0.219	<0.001	−
height minus MPH SDS at start	0.216	<0.001	−
height SDS at start	0.129	<0.001	−
BMI SDS at start	0.073	<0.01	+
father height SDS	0.05	<0.05	+
MPH SDS	0.046	<0.05	+
GH dose at start	0.036	<0.05	+
***ΔHt SDS during second year^b^***			
first-year ΔHt SDS	0.306	<0.001	+
maximum GH peak	0.301	<0.001	−
height minus MPH SDS at start	0.161	<0.001	−
height SDS at start	0.102	0.001	−
age at start	0.045	<0.05	−
***waning effect^c^***			
first-year ΔHt SDS	0.618	<0.001	+
age at start	0.161	<0.001	−
height SDS after first year	0.072	<0.01	+
height minus MPH SDS at start	0.065	<0.01	−
maximum GH peak	0.063	0.01	−
height SDS after second year	0.05	<0.05	+
height SDS at start	0.038	<0.05	−
BMI at start	0.037	<0.05	+

^a^gain in height SDS after 1 year of GH treatment; ^b^gain in height SDS after 2 years of GH treatment; ^c^waning effect, first-year ΔHt SDS minus ΔHt SDS during second year; GH, growth hormone; GHD, GH deficient; R², the coefficient of determination; pos, positive correlation; neg, negative correlation; MPH, mid parental height; BMI, body mass index.

### Response to GH Treatment During the Whole Treatment Period

**Figure 2:**
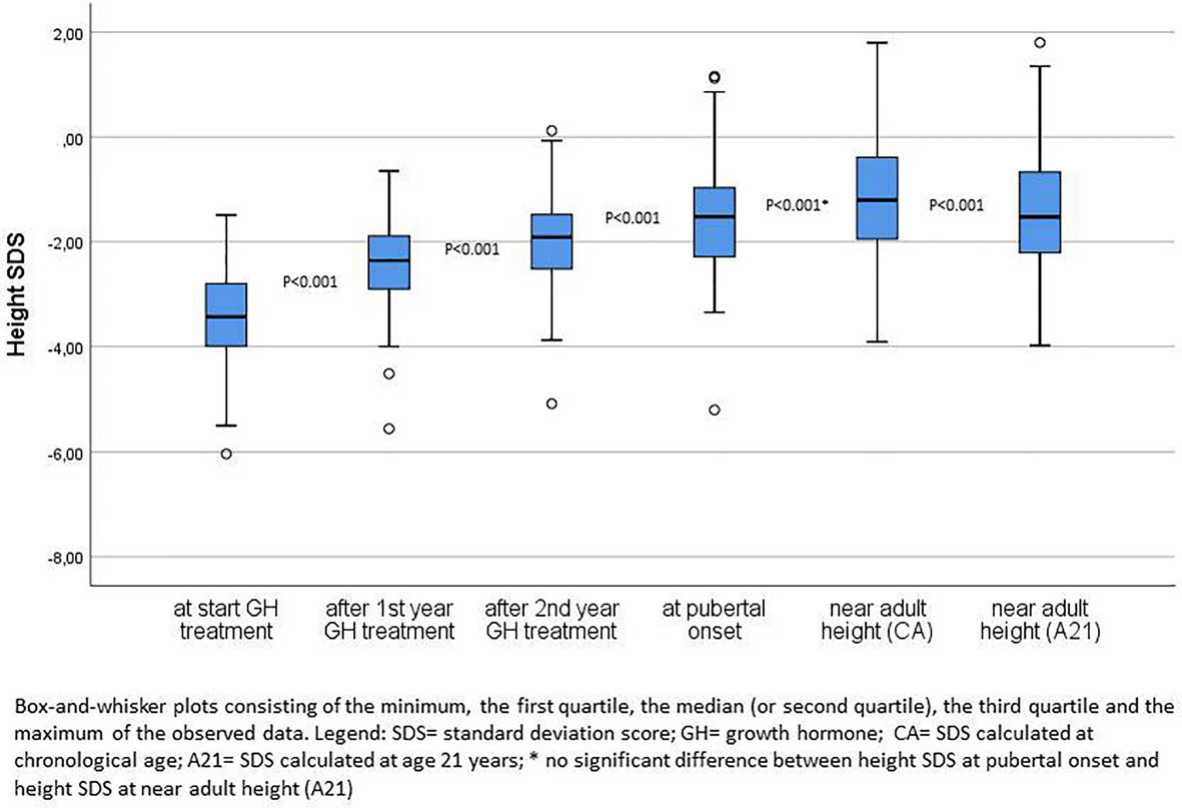
Height SDS at start of GH treatment, after first and second years of GH treatment, at pubertal onset and near adult height.

**Figure 2** compares the height SDS at start of GH treatment, after the first and second GH treatment years, at pubertal onset and at near AH. After one and two years of GH therapy, the median ΔHt SDS was respectively 1.03 and 1.44. At onset of puberty, median ΔHt SDS was 1.80. The median ΔHt SDS at nAH was 2.09 for chronological age (CA), but 1.86 when extrapolated to the age of 21 years (A21). The 2 year ΔHt SDS accounted thus for 69% (CA)–77% (A21) of the total increase in height SDS. Twenty five percent of the patients had a ΔHt SDS <1.0 at 2 years, 20% at pubertal onset, and 11% (CA)–16% (A21) at nAH.

After two years 46% of the patients had a height SDS <−2.0 and 35% at pubertal onset, whereas at the moment of nAH, 25% (CA) and 28% (A21) of the patients had a height SDS <−2.0. The median difference of the height SDS with the MPH SDS gradually diminished over time after the first and second prepubertal years of GH treatment until pubertal onset, respectively 1.34, 0.94, and 0.49 SDS. At start, 87% of the patients had a height − MPH SDS <−1.3, after two years this percentage decreased to 35%, and at pubertal onset it was 23% of the patients. Finally, 12% (CA) and 14% (A21) of the patients had a nAH − MPH SDS <−1.3.

### Response to GH Treatment in Isolated GHD *Versus* MPHD

Of 110 patients, 44 had multiple pituitary hormone deficiencies that were supplemented. ΔHt SDS after 1 year, ΔHt SDS after 2 years, and total ΔHt SDS were comparable between the group with isolated GHD and MPHD (**Table 3**).

**Table 3:** Response to GH treatment in isolated GHD *versus* MPHD.

	isolated GHD (n = 66)			MPHD (n = 44)			
	median	p25	p75	median	p25	p75	p-value
ΔHt SDS after 1 year^a^	1.04	0.66	1.36	1.02	0.61	1.59	n.s.
ΔHt SDS after 2 years^b^	1.40	1.07	1.99	1.47	0.82	2.07	n.s.
total ΔHt SDS (CA)^c^	2.14	1.59	2.97	2.04	1.53	3.20	n.s.
total ΔHt SDS (A21)^c^	1.88	1.20	2.69	1.81	1.14	2.79	n.s.

GH, growth hormone; GHD, growth hormone deficiency; MPHD, multiple pituitary hormone deficiencies; SDS, standard deviation score; ^a^gain in height SDS from start until after first-year GH treatment; ^b^gain in height SDS from start until after second year of GH treatment; ^c^gain in height SDS from start of GH treatment until nAH; CA, SDS calculated at chronological age; A21, SDS calculated at age 21 years; n.s., not significant.

### Prediction of a Poor Adult Height Outcome

**Figure 3:**
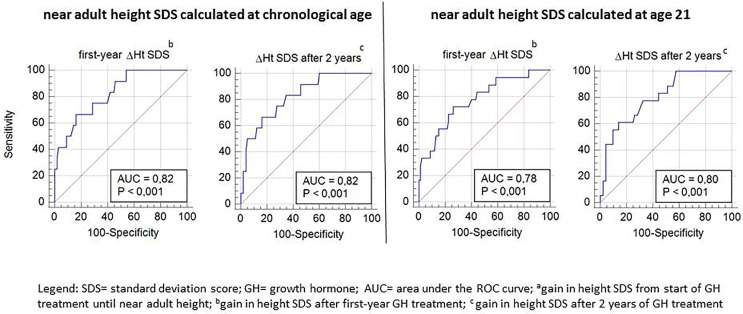
ROC curve analysis for Δ height SDS after the first and second prepubertal GH treatment years, with its sensitivity and specificity to predict total ΔHt SDS <1.

ROC curve analysis was performed for ΔHt SDS after the first and second prepubertal years of GH treatment in relation to the studied poor final outcome parameters (total ΔHt SDS <1, nAH SDS <−2, and nAH SDS − MPH SDS <−1.3). Only ROC-curves related to total ΔHt SDS <1.0 had an AUC ≥70% (varying between 78 and 82%) and were further analyzed (**Figure 3**).

**Tables 4A**, **B** show the cut-off values for ΔHt SDS after 1 and 2 prepubertal years of GH treatment, with their sensitivity and specificity to predict total ΔHt SDS <1.0 (CA and A21). The sensitivity to predict total ΔHt SDS <1.0 (CA) with a specificity of 95% was 42 and 50%, resulting in respectively 5/12 (4.6%) and 6/12 (5.5%) correctly identified poor final responders. At a 95% specificity level, 5/98 (4.5%) of the good final responders were misclassified as poor responders. If the SDS was calculated on age 21, the corresponding sensitivities were 33% after the first year and 44% after two years of GH treatment, giving respectively 6/18 (5.4% and 8/18 (7.2%) correctly diagnosed poor final responders. At a 95% specificity level, 5/92 (4.2%) of the good final responders were misclassified as poor responders. The ratio correctly diagnosed poor final responders/misclassified good final responders (1.0 at one year and 1.2 at two years) did not improve after two years of GH treatment.

**Table 4a:** ROC curve analysis: cut-off values for ΔHt SDS after 1 and 2 years of prepubertal GH treatment, with its sensitivity and specificity to predict total ΔHt SDS <1 (A21).

ΔHt after 1 year, SDS^b^	sensitivity (%)	specificity (%)	ΔHt after 2 years, SDS^c^	sensitivity (%)	specificity (%)
0.27	17	100	0.10	6	100
**0.47**	**33**	**95**	**0.72**	**44**	**95**
0.54	39	89	0.79	56	90
1.22	94	41	1.65	94	43
1.56	100	16	1.71	100	42
AUC: 78% (95% CI: 69–85%)			AUC: 80% (95% CI: 72–87%)		
n = 110 (P = 18 − G=92)			n = 110 (P = 18 – G = 92)		

GH, growth hormone; AUC, area under the ROC curve; CI, confidence interval. ^a^gain in height SDS from start of GH treatment until near adult height; ^b^gain in height SDS after 1 year of GH treatment; ^c^gain in height SDS after 2 years of GH treatment; A21, SDS calculated at age 21 years; n, number of patients; P, total ΔHt SDS <1; G, total ΔHt SDS ≥1.

Bold: values at 95% specificity.

**Table 4b:** ROC curve analysis: cut-off values for ΔHt SDS after 1 and 2 years of prepubertal GH treatment, with its sensitivity and specificity to predict total ΔHt SDS <1 (CA).

ΔHt after 1 year, SDS^b^	sensitivity (%)	specificity (%)	ΔHt after 2 years, SDS^c^	sensitivity (%)	specificity (%)
0.27	25	100	0.10	8	100
**0.41**	**42**	**95**	**0.64**	**50**	**95**
0.53	50	91	0.79	58	88
1.03	92	54	1.45	92	54
1.14	100	46	1.71	100	40
AUC: 82% (95% CI: 74–90%)			AUC: 82% (95% CI: 74–89%)		
n = 110 (P = 12 − G=98)			n = 110 (P = 12 – G = 98)		

GH, growth hormone; AUC, area under the ROC curve; CI, confidence interval. ^a^gain in height SDS from start of GH treatment until near adult height; ^b^gain in height SDS after 1 year of GH treatment; ^c^gain in height SDS after 2 years of GH treatment; CA, SDS calculated at chronological age; n, number of patients; P, total ΔHt SDS <1; G, total ΔHt SDS ≥1.

Bold: values at 95% specificity.

As shown in **Figure 1**, eight of 12 patients with a total height increase of <1 (CA) had both a below median ΔHt SDS after one and at two years of treatment.

## Discussion

The first year response to GH, in general represented as ΔHt SDS, is used by many clinicians to identify those children who may or may not benefit from long-term GH treatment. The first year response is also often used as a *post hoc* diagnostic criterion of GHD, especially in children with an idiopathic form of GHD. Although the first year response could be used to guide GH dosing, the current practice is to keep GH dosage stable over time on a body weight or body surface basis (in general between 0.25 and 35 µg/kg.day) in GHD patients, at least in Belgium and several other European countries (12–14). While the first year growth response to GH is highly associated with the adult height outcome, it is a weak predictor for a poor growth outcome on an individual basis, as we previously reported in a cohort overlapping the cohort of this study (6). In this study, we investigated if a lower waning effect in the second year might compensate for a lower first year response in prepubertal children with GHD, translating in a better predictability of a poor total height gain after two years of GH treatment.

A waning effect was observed in the majority of the patients in our study: only 3.6% of the GHD patients had a second year ΔHt SDS that was higher than the first year ΔHt SDS. The waning effect was greater in those with a more impressive height gain in the first year, which occurred more often in younger patients, in patients with a more severe GHD, and a greater height deficit in relation to their parental height. These results are consistent with previous studies in prepubertal patients with GHD (15, 16), with the exception of the absent association with the first year GH dose. The absent association with the GH dose in our study can be explained by the uniform dosing around 25 µg/kg.day. However, previous attempts trying to overcome this waning effect in GHD patients by modifying the dose or the frequency of the GH regimen have not been very efficacious, as the dose response relationship diminishes during the second year (17, 18).

We confirmed that the majority of the height gain in GH treated GHD children occurs during the first 2 years of treatment (19). In our study, 69% (CA) − 77% (A21) of the total height gain was obtained in the first 2 years. We showed that there was still some improvement in the percentages of children obtaining a normal height or a height within the expected target range after two years of treatment.

Despite its important contribution to the total height gain, the height increase after two years of treatment did not greatly improve the sensitivity to predict a poor growth outcome at the end of treatment: the sensitivity to detect with 95% specificity a poor total height increase at the end of treatment increased from 42% after one year to only 50% after two years of treatment. This finding can be explained by our observation that the waning effect observed during the second treatment year is in general lower in patients with a below average first-year response than in patients with an above average first-year response, explaining the only moderate correlation between the first year growth response and second year growth response. In most studies, the best predictor of the second year growth response was the first year response (16, 20). However, we observed that about a third of the above median first year growth responders grow slower than the median during the second treatment year. This might be explained among other factors by a declining adherence (21).

Despite the 8% (CA) − 12% (A21) increase in sensitivity for the second-year ΔHt SDS compared to first-year ΔHt SDS, the ratio correctly diagnosed poor final responders/misclassified good final responders did not change with a longer treatment duration due to the low (11–16%) prevalence of poor final responders. We hypothesize that predictability of poor final outcome will be better in a cohort with a higher prevalence of poor growth response, *e.g.* children born small for gestational age without catch-up growth or Turner syndrome. To illustrate this argument, if the poor response rate would have been 30% in this cohort (33 poor responders and 77 good responders) we would have identified 16 poor responders (13.6%) correctly and misclassified four good responders (3.6%)(at CA), a much better risk benefit ratio.

The patients remained short compared to their peers (mean nFAH SDS: −1.21 (CA) and −1.44 (A21) on Belgian references), but they almost reached their target height [nFAH minus MPH SDS: −0.19 (CA) and −0.43 (A21)]. This is consistent with other reports studying final height after GH treatment (19, 22–24).

This is the first study to evaluate the predictability of poor adult height outcome after two prepubertal GH treatment years in GHD children. This study has some shortcomings. Firstly, neither treatment adherence nor persistence of GHD was assessed routinely in the studied cohort. Secondly, the size of the cohort was rather small despite the national recruitment of patients. Near AH was taken as a proxy of AH as many patients usually stop GH treatment and disappear from follow-up when growth slows down to less than 2 cm per year and before AH is reached (25). To overcome this problem, nAH SDS could be calculated at a reference age of 21 years instead of the actual chronological age. This underestimates the real height SDS since most adolescents will still gain a few centimeters. On the other hand, since the mean height of the reference population also increases between 16 and 21 years, nAH SDS at the actual chronological age will overestimate the real height SDS. We therefore calculated nAH SDS both with age set at 21 years (worst case scenario) and at chronological age (best case scenario), accepting that the first method will underestimate and the second will overestimate the actual AH SDS.

In conclusion, the growth response after two prepubertal years of GH treatment did not meaningfully improve the prediction of poor near adult height outcome compared to the one year response. The decision to re-evaluate the diagnosis of GHD or adapt the GH dose in case of poor height response after 1 year should not be postponed for another year, as the prediction after two years has no added value in GHD children.

## Data Availability Statement

The raw data supporting the conclusions of this article will be made available by the authors, without undue reservation.

## Ethics Statement

This study was approved by the ethical committee of Brussels Free University and the University Hospital Brussels in Belgium. All subjects gave written informed consent in accordance with the Declaration of Helsinki.

## Author Contributions

SS, RR, and JS contributed to the conception and design of the study. SS organized the database. SS performed the statistical analysis. SS wrote the first draft of the manuscript. SS, RR, and JS wrote sections of the manuscript. All authors contributed to the article and approved the submitted version.

## Conflict of Interest

Author RR was employed by company Pendocon bv. RR has received consulting fees from Pfizer and Ferring and is a member of the Pfizer iGRO Advisory Board.

The remaining authors declare that the research was conducted in the absence of any commercial or financial relationships that could be construed as a potential conflict of interest.
